# DEPRESSION AND COGNITIVE FUNCTION IN OLDER MEXICAN DESCENT LATINOS: THE MEDIATING ROLE OF SOCIAL ENGAGEMENT

**DOI:** 10.1093/geroni/igae098.2970

**Published:** 2024-12-31

**Authors:** Liza Talavera-Garza

**Affiliations:** The University of Texas Rio Grande Valley, Edinburg, Texas, United States

## Abstract

Mexican descent Latinos have significantly higher rates of Alzheimer’s disease and related dementias (ADRD) compared to the general population. Latinos also report higher rates of depressive symptoms in older adulthood relative to non-Latino Whites. Depression in older age is associated with poor cognitive health; however, the mechanisms that underlie this relationship are not well-understood. The current study examined social engagement, which has been linked with better cognitive health, as a mediator of the association between depressive symptoms and cognitive function among older Mexican descent Latinos. Method: A convenience sample of Mexican descent Latinos (N=159), aged 55 to 88 years (M=68.2, SD=7.05) and without diagnosed cognitive problems, were recruited from a community in South Texas. They were administered in-person interviews, in their choice of English or Spanish in Summer 2023 that included demographic questions and measures of cognitive function, depressive symptoms, and social engagement.

**Results:**

Pearson correlations found that depressive symptoms were significantly negatively associated with social engagement (r = -.25, p <.002) and cognitive function (r = -.39, p <.001). Hierarchical Bayesian simulations indicated strong indirect effects of depressive symptoms on cognitive function through social engagement (unstandardized 95% CI = -.2408, -.0119, p<.0001).

**Conclusion:**

The current findings suggest that cognitive decline in individuals with depressive symptoms may occur because of reduced participation in social activities. Community-based approaches, with a focus on increasing social engagement in individuals who exhibit symptoms of depression, may be viable interventions that could be leveraged to reduce the burden of ADRD in Latinos.

